# The role of sagittal maxillary-mandibular relationships on perceptions of facial shape esthetics: A three-dimensional morphometric analysis

**DOI:** 10.1016/j.ajodo.2025.11.015

**Published:** 2026-01-14

**Authors:** Janick Decoste, Iacopo Cioffi, Marco F. Caminiti, Jose D. Aponte, Benedikt Hallgrímsson, Snehlata Oberoi, Siew-Ging Gong, Nathan M. Young

**Affiliations:** a Department of Orthodontics, Faculty of Dentistry, University of Toronto, Toronto, Ontario, Canada; b Department of Oral and Maxillofacial Surgery, Faculty of Dentistry, University of Toronto, Toronto, Ontario, Canada; c Department of Cell Biology and Anatomy, University of Calgary, Calgary, Alberta, Canada; d Department of Orofacial Sciences, University of California, San Francisco, San Francisco, Calif; e Department of Orthopedic Surgery, University of California, San Francisco, San Francisco, Calif

## Abstract

**Introduction:**

This study aimed to assess whether sagittal maxillary-mandibular skeletal relationships influence perceived facial esthetics in patients with varying sagittal skeletal discrepancies.

**Methods:**

We analyzed 3-dimensional facial models from cone-beam computed tomography scans of 40 patients (17 males and 23 females aged 14-48 years; ANB = 0° −10°), lacking skin color or hair, to isolate shape. A total of 100 laypeople rated these for esthetics and sexual dimorphism using visual analogue scales. Procrustes-based 3-dimensional geometric morphometrics quantified shape, and multivariate linear regression estimated vectors for visual analogue scales ratings. Linear regression tested correlations between shapes linked to the ANB angle and esthetics.

**Results:**

Perceived attractiveness was unrelated to age, asymmetry, or deviation from the population average shape. Esthetics correlated with lower facial third shape (r^2^ = 0.473, *P* = 0.058, 4.5% variation), particularly with anterior or inferior gnathion and pogonion displacement, with consistent patterns across sexes (r_v_ = 69.2°, *P* <0.0001). Shapes associated with higher ANB angles (indicating convexity) showed a negative association with attractiveness (r^2^ = 0.494, *P* <0.001). Sexual dimorphism was linked to cheek placement, facial roundness, and nasal tip position (r^2^ = 0.558, *P* = 0.001, 9.0% variation).

**Conclusions:**

Sagittal maxillary-mandibular relationships, as measured by the ANB angle and sagittal skeletal discrepancy, modestly influence facial esthetics, with straighter profiles and prominent chins rated more attractive. These findings suggest a partial biological basis for esthetic preferences, though individual and cultural factors, including smile esthetics and dental symmetry, also play a role. The study informs orthodontic treatment planning by highlighting the esthetic impact of jaw alignment.

Facial attractiveness significantly influences the quality of life, with more attractive individuals often experiencing better social and professional outcomes.^1^ But what makes a face more or less attractive? Although some have suggested the existence of universal beauty standards that reflect deep geometric or mathematical principles,^2^ most studies find that perceptions of attractiveness vary widely, shaped by individual preferences, cultural norms, and temporal trends.^3^ This variability raises questions about whether any universal principle can explain facial esthetics or if, instead, preferences are largely subjective and prone to individual idiosyncrasies.^4^

Evolutionary theory suggests that esthetic preferences may co-evolve with biological traits that improve reproductive success or signal individual fitness. For example, Darwin proposed that some traits may evolve simply because they appeal to the observer (ie, they influence mate choice).^5^ Such attractive traits may in fact be honest signals of an individual’s overall fitness, such as how a preference for bilateral symmetry may reflect an individual’s developmental stability, but preferred traits need not be functional and may even be costly to survival (eg, elaborate ornamentation). In either case, observer preferences act as a form of selection, and if heritable, increase the likelihood that both the preference and the trait will be reproduced in the next generation.^6^ That said, distinguishing whether traits perceived to be attractive evolve because of selection on the heritable preferences of the observer, enhanced fitness in individuals with those traits, or a mix of both, remains challenging.^7^

In this regard, the relationship of esthetics and facial shape to sagittal skeletal discrepancy (SSD) provides a potentially useful test of these questions. Although mild SSD often has minimal functional impact, increasingly large discrepancies lead to jaw size mismatch that may affect mastication, speech, sleep, and dental health.^8^ Adults with SSD often cite facial appearance as their primary concern,^9^ hinting at a link between sagittal jaw alignment (ie, function) and self-perceived esthetics (ie, preference).^10^ Self-perception of smile esthetics also plays a critical role in patient satisfaction,^11^ with smile symmetry influencing overall facial harmony and self-esteem in adolescents and adults undergoing orthodontic treatment.^12^ Unsurprisingly, clinicians understand that by normalizing maxillary-mandibular relationships across all planes, they will help to both improve occlusion and foster facial harmony.^13^

To explore the 3-dimensional (3D) shape correlations of facial esthetics and their relation to SSD, this study uses previously collected cone-beam computed tomography (CBCT) scans, population-averaged visual analog scale (VAS) ratings, and 3D geometric morphometrics (3DGM). Unlike 3D facial scans that include skin color or hair—potential esthetic confounders—CBCT isolates soft and hard tissue morphology without these features, allowing for a more direct assessment of shape alone.^14^ VAS provides a simple analog approach to quantification,^15^ whereas 3DGM improves upon studies using 2-dimensional (2D) linear or angular methods, such as silhouettes or photographs, as it enables the quantification and visualization of full shape variation of the sample.^3,16-18^ We hypothesize that perceived facial attractiveness significantly correlates with jaw-related morphologic features, specifically the sagittal skeletal relationship (measured by the ANB angle), independent of sex, perceived masculinity or femininity, age, or asymmetry. Alternatively, we tested if attractiveness aligns with the average population shape (ie, whether preferences align with avoidance of extremes).^6^ This research addresses a gap in understanding how jaw alignment influences esthetic perceptions, potentially informing evidence-based orthognathic surgery planning for SSD.

## MATERIAL AND METHODS

We analyzed 440 archival CBCT scans from the University of California, San Francisco (UCSF) School of Dentistry, collected from 2004 to 2007, for planning and diagnostic purposes under informed consent and institutional review board (IRB) approval from all participants for use in research, as previously published.^19^ These imaging data were subsequently approved for additional archival research (UCSF IRB number: 11-06996), as described by Young et al^20^ The scans were originally acquired using a MercuRay CBCT scanner (Hitachi Medical, Tokyo, Japan) with an estimated radiation exposure of approximately 200 mSv. Participants were seated upright, with the x-ray tube and imaging screen rotating around their heads. They were instructed to remain still, keep their teeth in occlusion, lips relaxed, avoid swallowing, and maintain their tongue against the palate with their head in a natural position. Scanner settings were 110 kVp and 10 mA, producing 512 slices in a 10-second scan, with a 19 × 19 × 19 cm field of view (FOV) and a voxel size of 0.38 mm. Images were reconstructed using CBWorks (version 2.1; Cyber Med, Seoul, Korea) and Avia (Hitachi Medical Devices, Tokyo, Japan) software and saved in digital imaging and communications in medicine format. Each scan included the participant’s ANB angle classification, sex, and age, with all other personal information anonymized.

The CBCT scans were collected at a time when large-FOV CBCT was more commonly accepted for diagnosis. It was believed that because CBCT reduced distortion of craniofacial imaging, temporomandibular joint, airways, and facial soft tissue, it would also improve measurement and treatment planning. Moreover, the risk to benefit ratio was an active area of research, and this approach was considered within safe limits per the Declaration of Helsinki.^21^ However, with the benefit of hindsight and additional information, current as low as reasonably achievable (ALARA) guidelines favor even lower-dose alternatives, such as conventional 2D radiographs for routine orthodontics, much reduced FOV, or use of ultra-low dose CBCT, when warranted. We acknowledge that current ALARA principles prioritize lower-dose imaging (eg, conventional radiographs) for routine orthodontics, particularly for young patients, because of a greater appreciation of their increased sensitivity to ionizing radiation. Guidelines from the American Academy of Oral and Maxillofacial Radiology and recent reviews emphasize that although large-FOV CBCT should not be routine, the use of archival datasets—when IRB-approved and justified—aligns with ethical research practices to derive value from past exposures without promoting outdated protocols.^22,23^ This study is consistent with these guidelines. We use archival data to analyze facial shape alone, and not to endorse historical imaging protocols. The current study received approval from the UCSF IRB (IRB number: 11-06996) to access and study this archival data expressly to maximize the usage of a now irreplaceable dataset. The findings remain relevant to understanding esthetic perceptions, independent of imaging methods.

**Fig 1.**
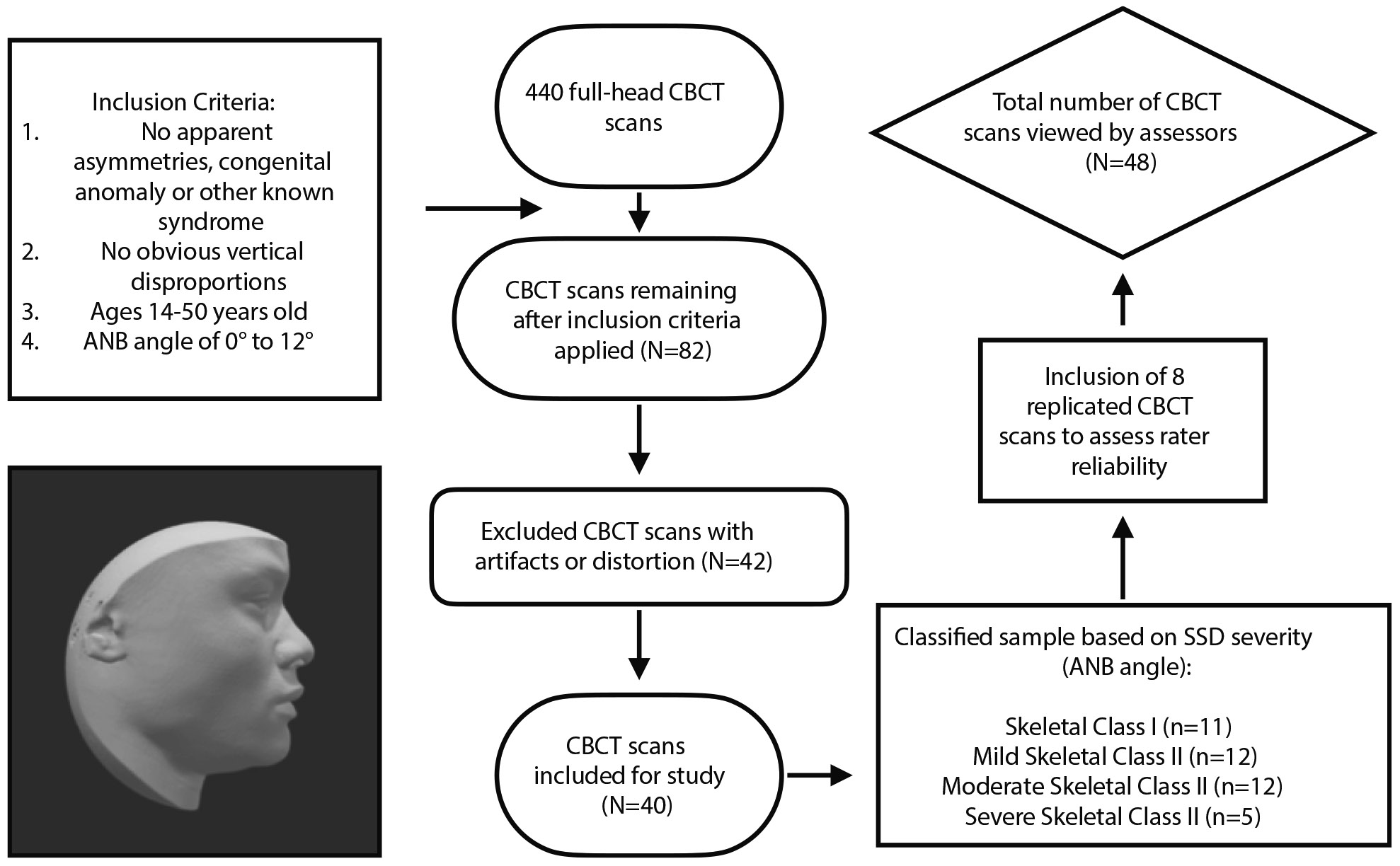
Inclusion and exclusion criteria. Flow chart illustrating sample generation for esthetic rating. A snapshot of a video clip shown to assessors is included (*bottom left*).

From the initial pool of 440 scans, 82 were selected based on the following inclusion criteria (Fig 1): (1) no apparent facial asymmetries, congenital anomalies, or known syndromes; (2) no significant vertical disproportions in the lower face, defined by the Eastman normal value for lower anterior face height or total anterior face height (55%) with a standard deviation of ±2%;^17^ (3) age range of 13-50 years at the time of scanning, and (4) ANB angle of 0° −10°, measured via Steiner cephalometric analysis on reconstructed lateral cephalograms.

Scans were grouped by the ANB angle^17^: (1) orthognathic: 0° to <3.6° (n = 11; 6 females and 5 males); (2) mild SSD: ≥3.6° to <6° (n = 12; 7 females and 5 males); (3) moderate SSD: ≥6° to <8° (n = 12; 6 females and 6 males); and (4) severe SSD: ≥8° (n = 5; 3 females and 2 males).

To ensure a balanced distribution and limit the assessment time to 30-40 minutes, 40 CBCT scans were randomly selected from each SSD category (Fig 1). In addition, 8 scans (20% of the sample) were duplicated to test rater reliability. Six scans were found to have imaging artifacts, so they were randomly replaced with an additional 6 scans. The final sample consisted of 48 CBCT scans from 40 patients: orthognathic (n = 11), mild SSD (n = 12), moderate SSD (n = 12), severe SSD (n = 5), and duplicates (n = 8; 2 per SSD category) (Fig 1). This sample included 40 participants (22 females and 18 males), with a mean age of 22.3 years (range: 14-48 years) and a mean ANB angle of 5.1° (range: 0.1° −10.6°).

For each selected CBCT scan, a 3D model of the external soft tissue surface was generated by thresholding in Amira 3D software (Mercury Software, San Francisco, Calif), as described by Young et al^20^ One operator (J.D.) reviewed all models in MeshLab (version 1.2.1; Institute of Information Science and Technologies, Pisa, Italy) to identify imaging artifacts or distortions, excluding further 6 scans because of poor resolution.

A group of laypeople aged 18+ years, with no dental knowledge or prior experience evaluating facial esthetics or sexual dimorphism, was recruited. An a priori power analysis (G*Power3)^24^ assumed a small effect size (*d* = 0.15), α = 0.05, and power = 0.90, requiring 99 raters. We recruited 108 potential assessors, who completed a demographic questionnaire and the State-Trait Anxiety Inventory questionnaire^25^ to assess psychological status, as anxiety can influence attractiveness judgments.^26^ Assessors with State-Trait Anxiety Inventory questionnaire scores >52 (indicating potential anxiety disorder) were excluded.^26^ Three were excluded for high trait anxiety and 5 for incomplete scoring, leaving 100 assessors. This group was primarily self-identified white Canadians (53%), with bachelor’s degrees (81%) and a mean age of 30 years (range: 19-65 years). Of these, 61 were first-year dental students, and 39 had diverse backgrounds. Mean state and trait anxiety scores were 31.3 (± 8.4) and 34.4 (± 8.2), respectively, indicating no clinically significant anxiety.

Assessors were divided into groups of 15-20 and evaluated the 3D models in a quiet seminar room. For each model, a standardized video clip was created, showing the face rotating around a vertical axis (aligned with the Frankfort horizontal plane) from right to left profile, with a pause at the frontal view. These clips, mimicking real-life perception, were projected in random order on a 120-in × 90-in screen using Power-Point 2016 (version 16.36; Microsoft, Redmond, Wash). Assessors rated each face for facial attractiveness and sexual dimorphism using a 100-mm VAS with anchors: extremely unattractive to extremely attractive for esthetics and masculine to feminine for dimorphism.^15^ They had 20 seconds to view and rate each clip, with 5-second transitions, completing the process in approximately 40 minutes. No talking, eating, or drinking was allowed, and assessors could not revisit prior clips. Scores were measured (J.D.), averaged per model, and analyzed using descriptive statistics and intraclass correlation coefficients to assess rater reliability.

**Fig 2.**
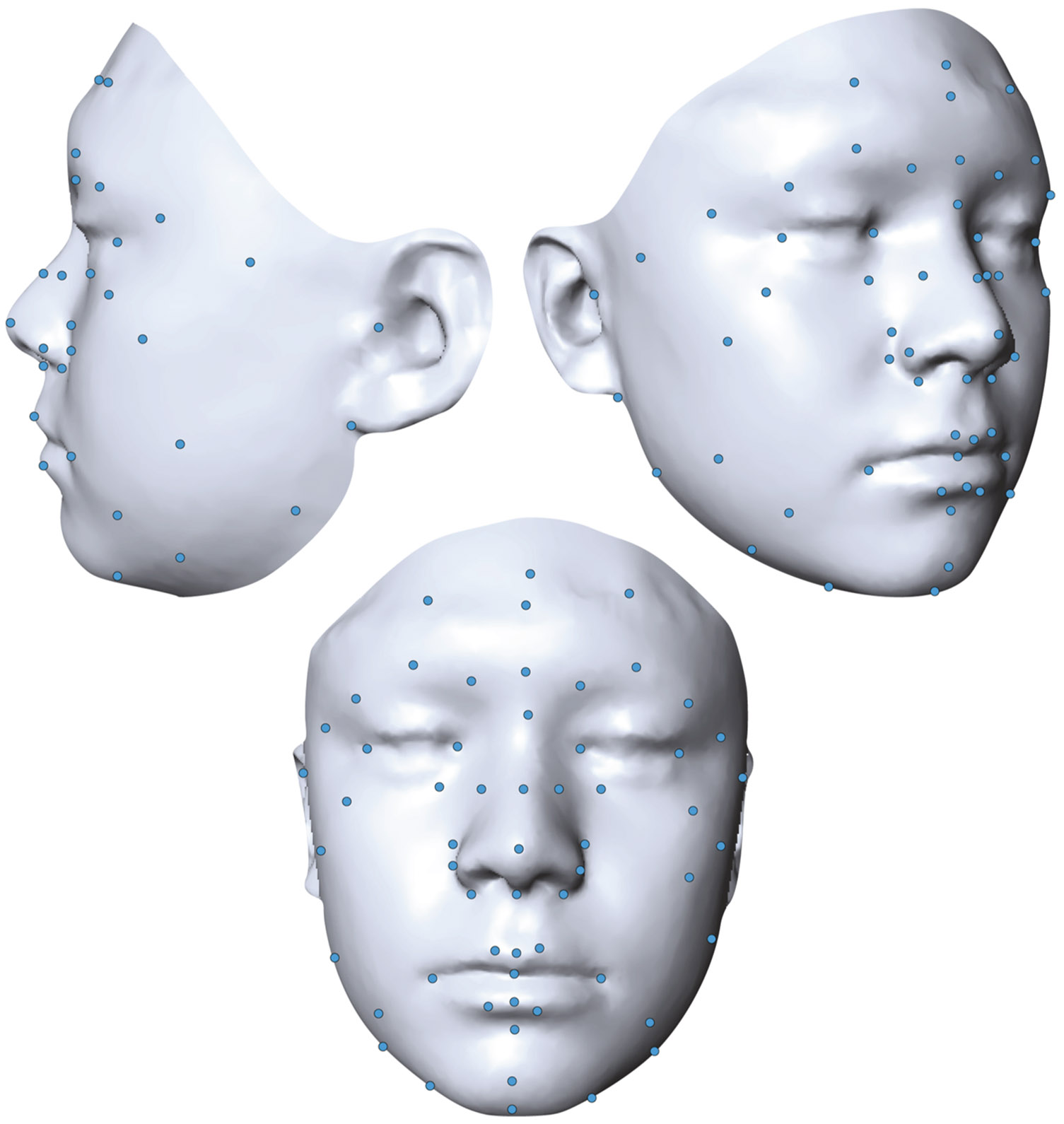
Soft tissue landmarks (lateral, angle, and frontal view). Landmarks (*blue*, n = 65) generated using Hallgrímsson et al^27^ from an atlas-based automated registration (lateral, angle, and frontal views).

Shape data were derived using a semi-automated landmarking method (Fig 2; Hallgrímsson et al^27^). An atlas was created from 1 scan, cropped, and decimated to approximately 2,500 points for computational efficiency. This atlas was nonlinearly registered to 10 random scans, then to all scans, using the optimal step nonrigid iterative closest point algorithm. Sixty-five 3D (x, y, and z) landmarks were calculated (Fig 2; Hallgrímsson et al^27^). Procrustes superimposition aligned these landmarks into a common shape space by scaling to a centroid size of 1, rotating to minimize squared deviations, and centering on the group mean.^16^ Asymmetry was assessed by calculating deviations of left-right paired landmarks from midline landmarks, yielding symmetrical and asymmetrical components. Procrustes distances from the population mean were computed, in which lower values indicated closer alignment to the average shape, to test whether attractiveness correlates with proximity to the mean.

Principal components analysis was used to identify major shape variation axes in the full sample (n = 440), with analysis of variance testing for shape differences between the full and rated samples (n = 40). Multivariate regressions examined relationships between Procrustes data (and asymmetry) and continuous variables: facial attractiveness, sexual dimorphism, age, and Procrustes distance. Independent variables included size, age, and VAS scores; dependent variables were Procrustes or asymmetry data. Significance was assessed via permutation (1000 replicates). Regression coefficients quantified shape-variable relationships, and linear regression tested associations with ANB angle, age, and VAS scores for attractiveness and sexual dimorphism. Shape vectors were visualized by warping an average face (minimum Procrustes distance) into extreme configurations using a thin-plate spline algorithm in Landmark Editor (University of California Davis, Davis, Calif). Analyses were performed in MorphoJ (version 1.08.02; Klingenberg^28^, Chris Klingenberg, Zurich, Switzerland).

## RESULTS

**Fig 3.**
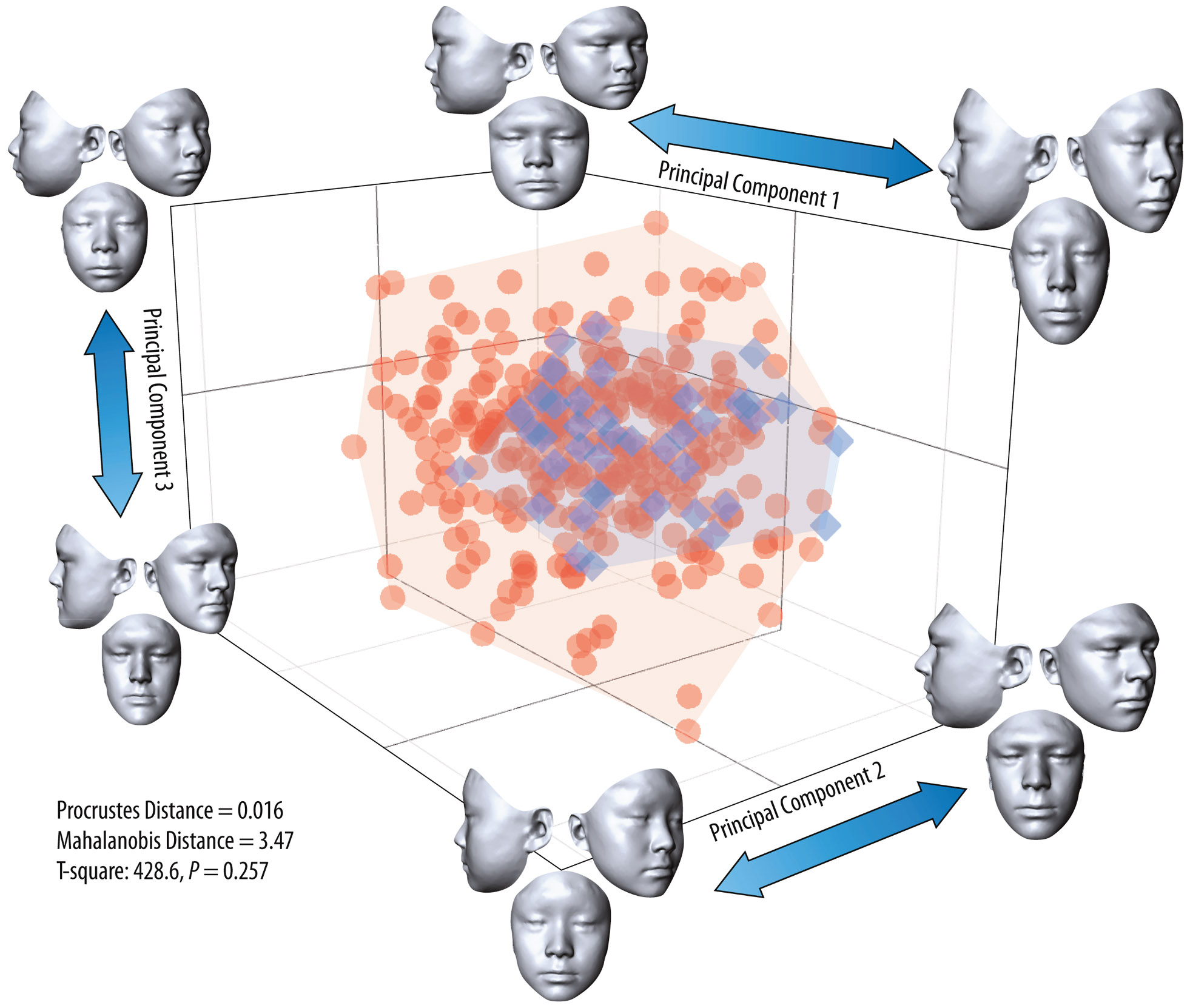
Principal components analysis of facial shape. PC1-3 of shape variation ordination for the total sample (*red*, n = 324) and assessed sample (*blue*, n = 40). Each axis shows the population-calculated mean shape warped to the extreme using the associated eigenvector (lateral, angle, and frontal views). *PC1-3*, Principal components 1-3.

**Fig 4.**
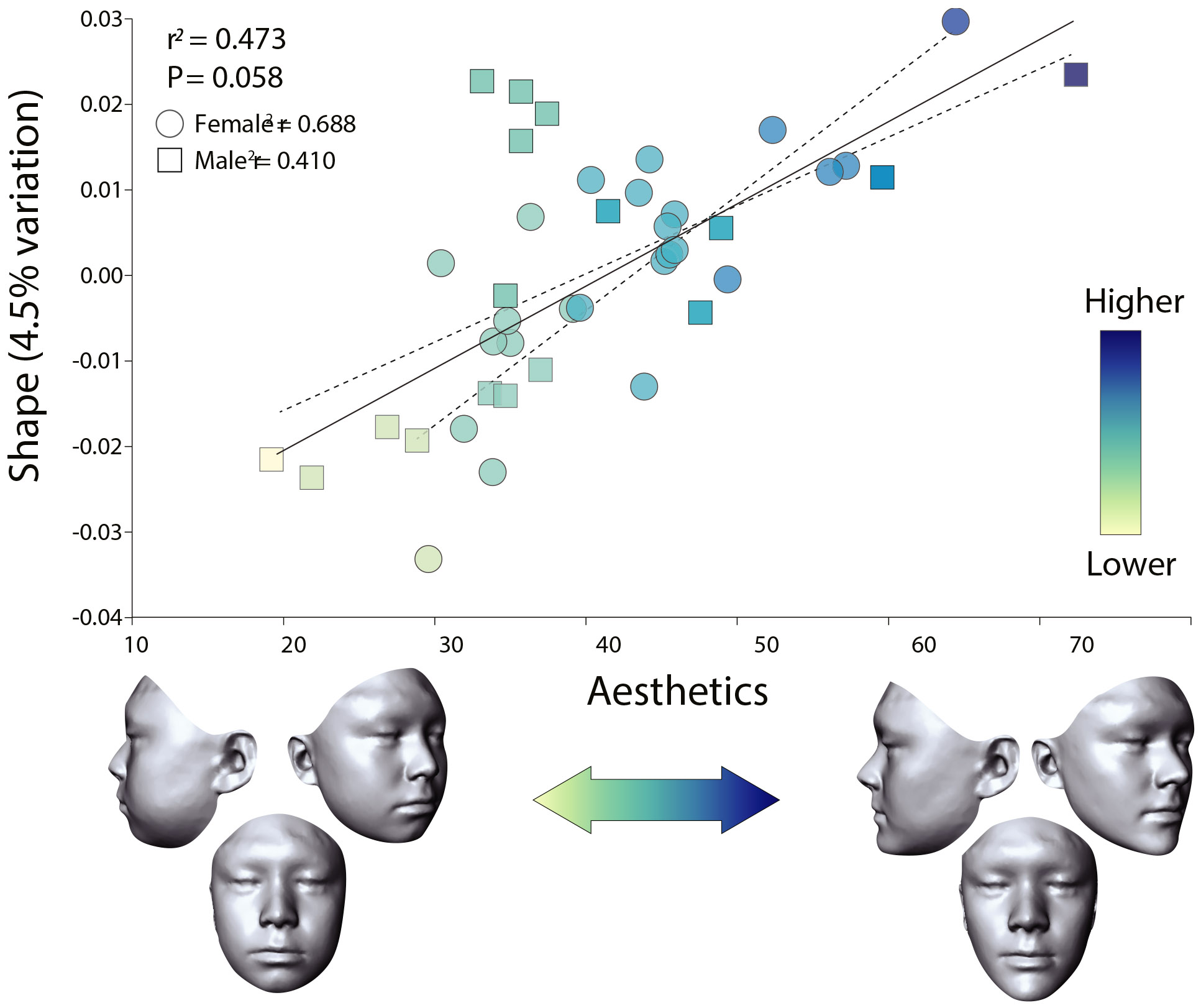
Multivariate regression of averaged perceived esthetics on shape. Population-averaged esthetic scores predicted 4.5% of facial shape variation (r^2^ = 0.473, *P* = 0.058). Faces represent the sample mean warped along the shape vector to extreme values. More attractive faces have prominent chins and vertical profiles; less attractive faces have retrusive chins and convex profiles. Sex-stratified vectors for females (*circles*, r^2^ = 0.688, *P* <0.0001) and males (*squares*, r^2^ = 0.410, *P* <0.0001) are shown.

**Fig 5.**
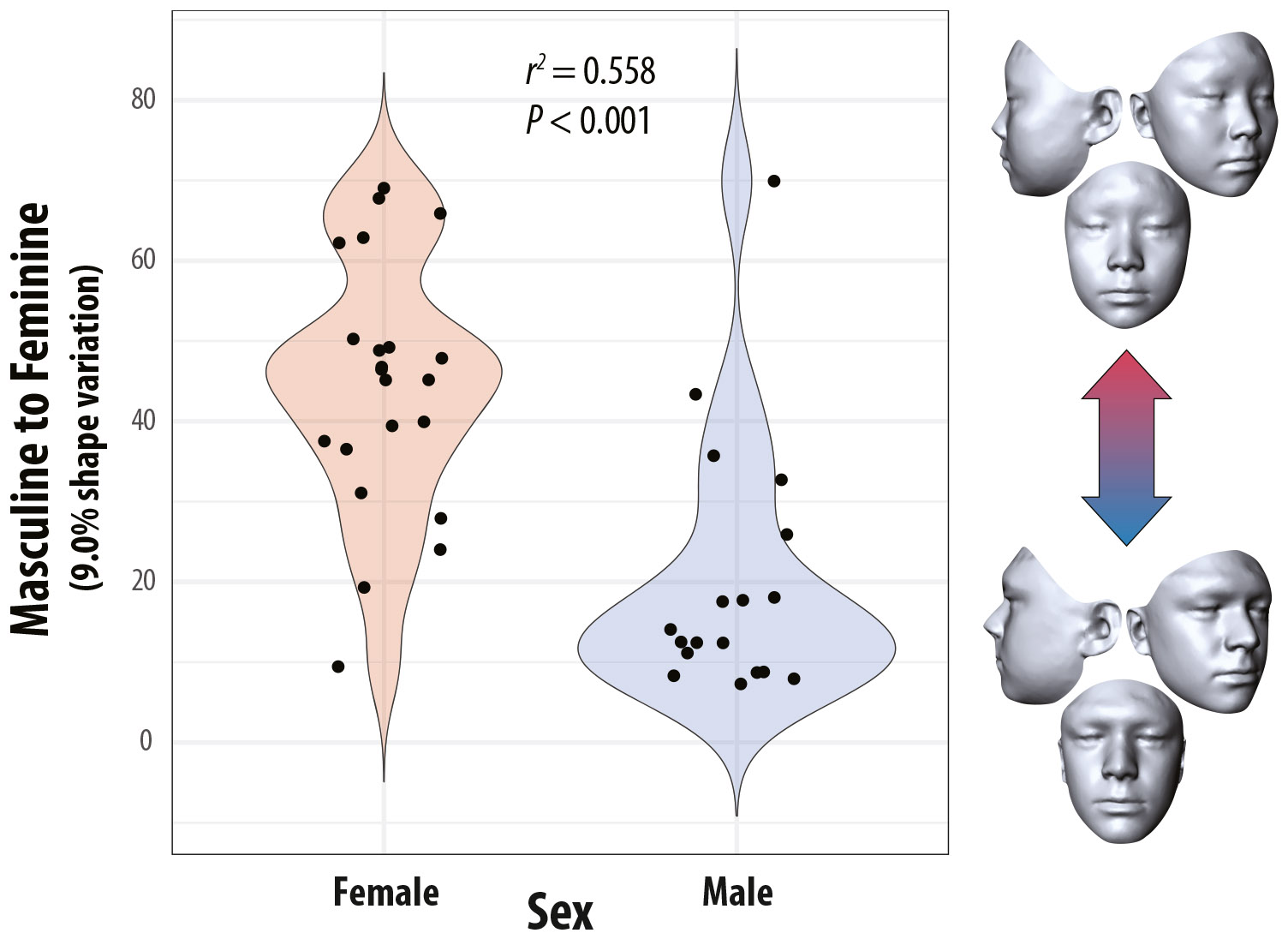
The distribution of perceived femininity to masculinity shapes scores binned by actual biological sex. Multivariate regression of femininity to masculinity perceptions on shape shows overlap across sexes despite significant mean differences.

**Fig 6.**
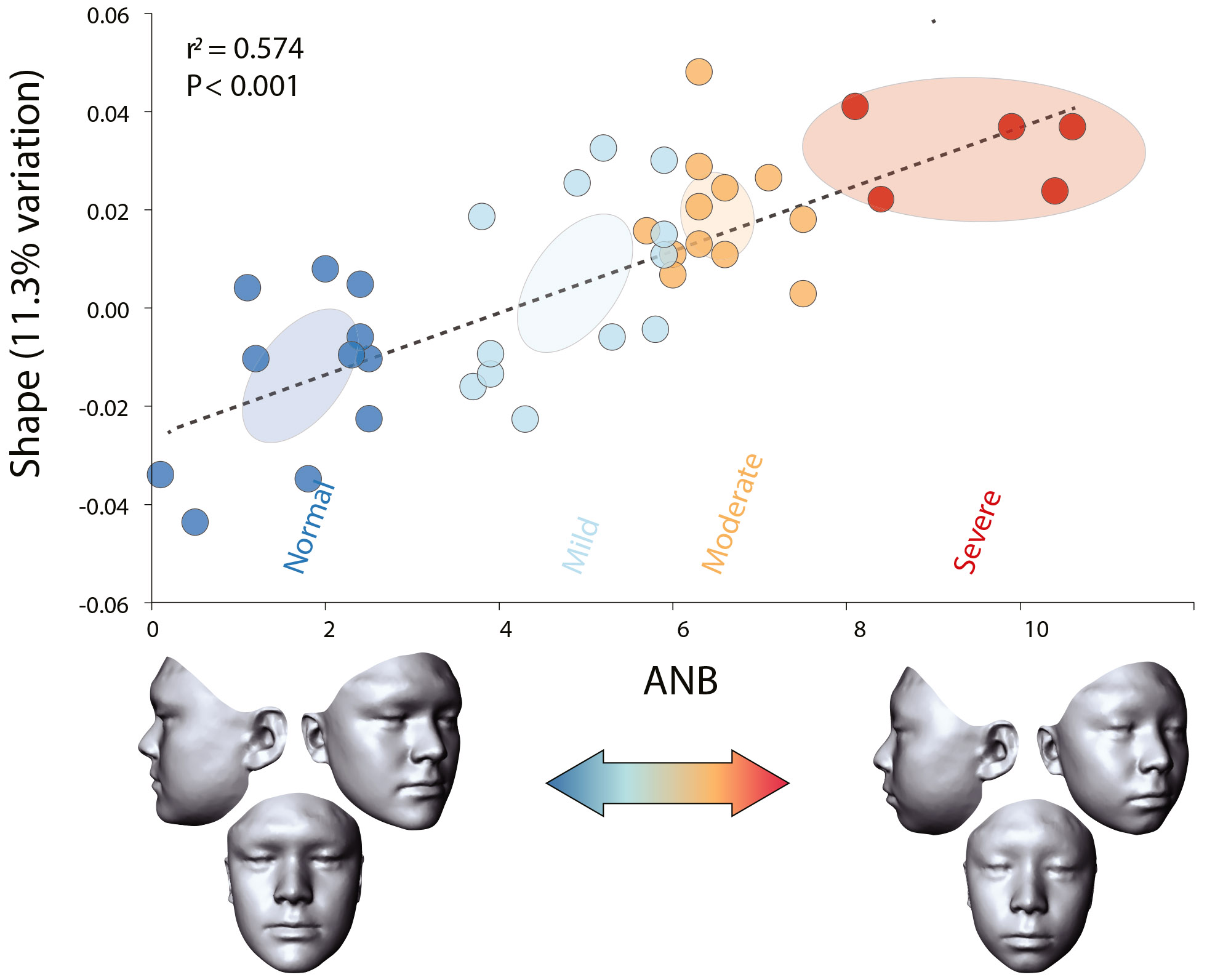
Multivariate regression of ANB on shape. ANB predicted 11.3% of total shape variation (*P*<0.001). Higher ANB, as independently measured from 2D radiographs, is associated with a retrusive chin and severe SSD in 3D CBCT.

**Fig 7.**
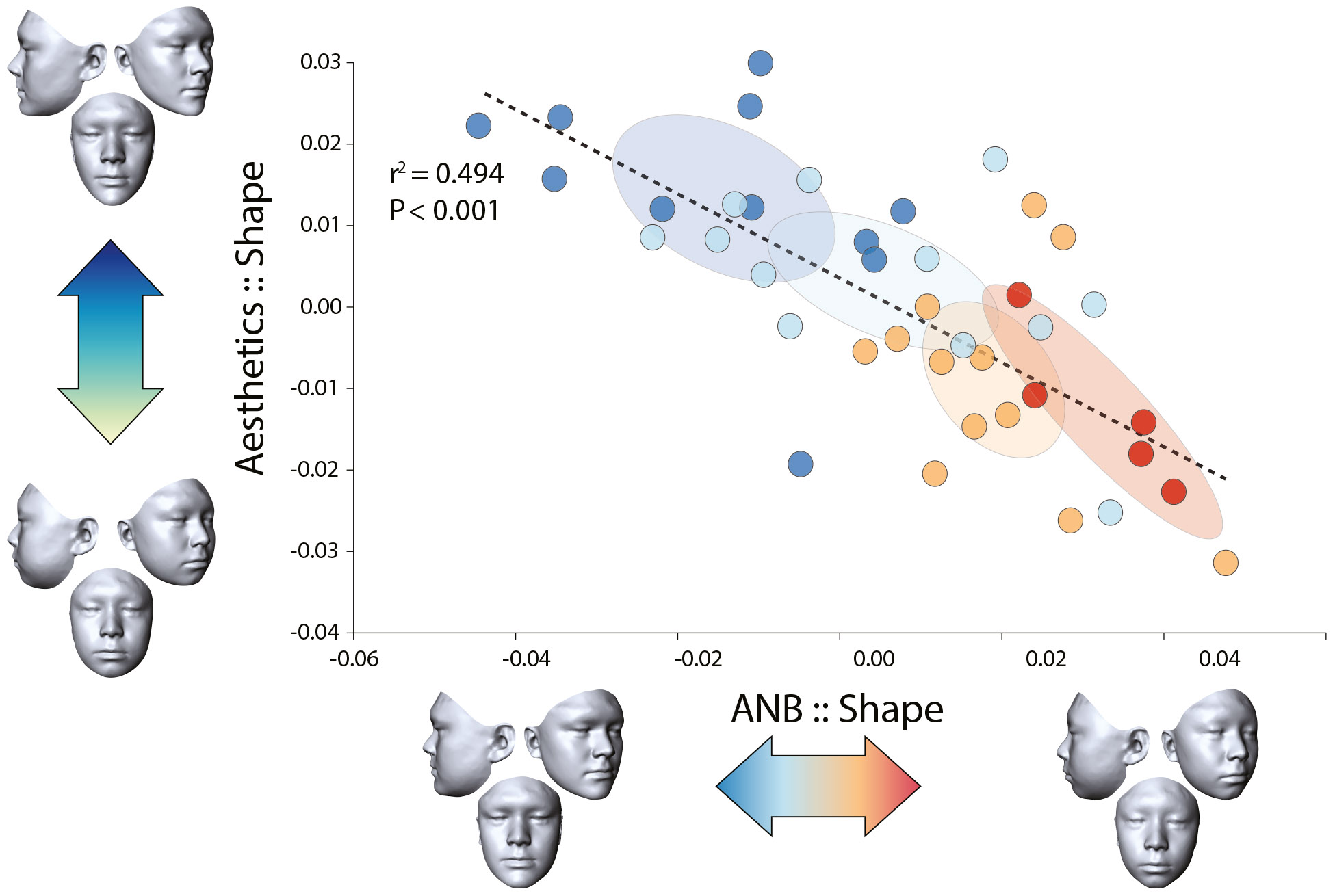
Linear regression between ANB shape scores and esthetic shape scores. Multivariate regression scores of ANB shapes and esthetic shapes are significantly associated (r^2^ = 0.494, *P* <0.001), supporting the hypothesis that jaw alignment influences esthetic perceptions of the face.

The rated subsample (n = 40) showed no significant shape difference from the overall sample (n = 324, Procrustes distance = 0.002, Hotelling’s T^2^ = 0.27, *P* = 0.257; Fig 3). Perceived facial attractiveness was unrelated to age (r^2^ = 0.091, *P* = 0.604), asymmetry (*P* = 0.131), or Procrustes distance from the mean (r^2^ = 0.028, *P* >0.05). However, shape correlated with attractiveness (r^2^ = 0.473, *P* = 0.058, 4.5% variation; Fig 4) and sexual dimorphism (masculinity or femininity) (r^2^ = 0.558, *P* <0.001, 9.0% variation; Fig 5). ANB angle influenced shape (r^2^ = 0.574, *P* <0.001, explaining 11.3% of variation; Fig 6) and was negatively associated with attractiveness-related shapes (r^2^ = 0.494, *P* <0.001; Fig 7).

Attractiveness was linked to lower facial third shape, including anterior or caudal gnathion and pogonion, cranial labiale inferius and cheilion, and reduced philtrum and labiale superius projection. Straighter profiles with prominent chins were rated highly, whereas convex profiles with retrusive chins scored lower (Fig 4). Consistent with these findings, shapes linked to higher ANB angles (indicating convexity) showed a negative association with perceived attractiveness (Fig 7). Sex-stratified analyses showed stronger associations in females (r^2^ = 0.688, *P* <0.0001) than males (r^2^ = 0.410, *P* <0.0001), but shape vectors were correlated across sexes (angle = 69.2°, r_v_ = 0.355, *P* = 0.0001), indicating that similar shape factors influence perceived attractiveness across sexes. Sexual dimorphism involved cheek size, nasal tip, chin, and lower lip displacement, with consistent patterns across sexes (Fig 5).

## DISCUSSION

This study used 3DGM to explore how sagittal maxillary-mandibular relationships influence facial esthetics in an orthodontic population. In contrast to prior studies that relied on linear and angular measurements,^29^ we analyzed the entire facial surface in 3D, enabling detailed visualization of shape variations linked to attractiveness and dimorphism. Rather than using highly attractive individuals (eg, models or actresses),^30,31^ our sample comprised a general population of orthodontic patients. We recruited laypeople as raters to ensure naïve assessments based solely on personal experience, not professional training. We did not control for ethnicity or age in either the subjects (CBCT scans) or raters, as prior research suggests esthetic judgments are consistent across cultural backgrounds.^32^ Raters were given 20 seconds per image to view and score, consistent with Stróżak and Zielińska^33^ Our findings revealed associations between facial attractiveness, sexual dimorphism, and morphologic features of the lower facial third, with the sagittal maxillary-mandibular relationship (ANB angle) emerging as a modest shared determinant of attractiveness across raters, consistent with prior findings.^34-36^ Straighter profiles with prominent chins were rated highly, aligning with Kanavakis et al,^9^ who linked specific facial shapes to self-perceived attractiveness. However, as ANB is a shape measure and our design emphasized sagittal relationships, its role should not be overstated. Other facial dimensions, such as vertical proportions or soft tissue thickness, may also contribute to esthetics, as shown in laser facial scanning studies.^37^

The lower facial third, particularly the chin and lips, is widely recognized as critical to facial beauty.^36-38^ The chin’s shape contributes harmony, character, and personality.^38,39^ Straight profiles are generally rated more attractive than convex or concave ones, with a chin positioned within the lower third and sagittal central fifth deemed most appealing.^40^ Although these traits align with clinical assessments of SSD, previous studies found no significant link between ANB values and facial attractiveness.^41,42^ In contrast, our study identified a modest relationship between lower face soft tissue esthetics and SSD. Positive perceptions of attractiveness were associated with a more anterior position of hard and soft tissue pogonion, suggesting a normalized or slightly reduced ANB.^43^ Consistent with Naini et al,^39^ the most attractive facial vector aligned the chin with the 0^o^ meridian (a vertical line from soft tissue nasion perpendicular to the Frankfort horizontal plane; Gonzalez-Ulloa^44^). These differences may reflect the enhanced sensitivity of our 3D morphometric approach, despite its modest contribution to overall esthetic variation. These lower face features also intersect with dental esthetics, as orthodontic corrections of SSD can enhance smile symmetry and gingival display, which are key contributors to perceived attractiveness.^45,46^

Facial profile and chin morphology also influence perceptions of sexual dimorphism.^33,47^ In females, a slightly convex profile is often considered more attractive,^48^ whereas in males, a straight profile with a prominent, broad chin is preferred.^49^ Our results align with these findings, showing a retrusive chin as more feminine, though its effect on esthetics was less pronounced than the nose’s position. A retrusive nose was linked to increased femininity, consistent with protrusive noses being rated masculine and retrusive ones feminine.^45,50^ Prominent, rounded cheeks were also associated with femininity, corroborating studies that link enlarged cheeks to feminine appearance.^51^ Modifying the nose, cheeks, and, to a lesser extent, the chin significantly altered perceived masculinity and femininity, consistent with prior research.^31,47^ Sexual dimorphism in smile components, such as buccal corridor width and tooth display, further modulates these perceptions, with recent studies showing consistency across genders in orthodontic populations.^52^

The archival CBCT data, collected from 2004 to 2007, were originally obtained under informed consent for research purposes, with a focus on understanding the potential benefits of newer volumetric imaging modalities relative to traditional 2D radiographs.^19^ Although deemed safe at the time, current ALARA guidelines favor lower-dose alternatives for routine orthodontics. This historical context limits direct application to modern practice but does not invalidate this study’s findings, which focus on shape rather than imaging protocols. Moreover, previous studies of this dataset directly contributed to growing appreciation that other volumetric modalities may be sufficient to characterize skeletal shape without ionizing radiation (eg, 3D photography), highlighting the importance of continued analysis of legacy datasets.^20^

The broad age range (14-48 years) may introduce morphologic variability, though no significant age-attractiveness correlation was detected in this dataset. The small severe SSD subgroup (n = 5) likewise limits statistical power for this category. Restricting lower face height to ± 1 standard deviation may reduce generalizability by excluding natural variability. The lack of hair and skin color in CBCT models may have influenced layperson ratings, as these features affect real-world perceptions. Socioeconomic factors, such as access to orthodontic care, may influence esthetic perceptions too, as symmetry and alignment are often associated with higher social status.^13^ Patient-centered outcomes, such as smile esthetics, are increasingly prioritized in treatment planning, highlighting the need for studies like ours.^11^ Future research should integrate dynamic assessments, such as smile analysis, using modern low-dose imaging to explore how dental symmetry interacts with sagittal relationships.^53,54^ Future studies would also benefit from exploring additional vertical and transverse facial dimensions, incorporating diverse populations, and using modern imaging protocols.

## CONCLUSIONS

This study demonstrates that sagittal maxillary-mandibular relationships, as measured by ANB angle and SSD, modestly influence perceived facial esthetics, with straighter profiles and prominent chins rated more attractive. These features also contribute to sexual dimorphism, particularly in females. Although SSD accounts for a small fraction of shape variation, its esthetic impact informs orthodontic and orthognathic treatment planning, balancing function and appearance. Future research should explore additional facial dimensions and modern imaging protocols to enhance generalizability.
